# Integrated Transcriptomic and Developmental Analyses Provide Insights into the Intrafloral Stamen Differentiation in *Cassia fistula* L.

**DOI:** 10.3390/plants14223490

**Published:** 2025-11-15

**Authors:** Zhonglai Luo, Tingting Duan, Xiaoyuan Li, Jianxuan Zhou, Qiankun Liu, Libo Jiang

**Affiliations:** 1 School of Life Sciences and Medicine, Shandong University of Technology, Zibo 255000, China; 2 College of Coastal Agricultural Sciences, Guangdong Ocean University, Zhanjiang 524088, China

**Keywords:** *Cassia fistula*, flower development, heteranthery, stamen differentiation, transcriptomics

## Abstract

Selective pressure targeting male functions plays a crucial role in the evolution of floral morphological traits. In some angiosperm groups, flowers contain two or more sets of stamens that vary in size, color, and morphology, a phenomenon known as heteranthery. This reflects an evolutionary adaptation of stamens. However, the developmental basis and molecular mechanisms remain poorly understood. This study integrates transcriptomic and developmental approaches to elucidate the molecular and morphological mechanisms underlying intra-floral stamen differentiation in *Cassia fistula* L., an economic leguminous tree exhibiting heteranthery with three distinct stamen types: long stamens (LS), short stamens (SS), and degenerated stamens (St). We documented asynchronous stamen primordia initiation and development trajectories across stamen types. Transcriptomic profiling and protein–protein interaction analysis identified differentially expressed genes (DEGs) between filaments of the three stamen sets, with significant enrichment in brassinosteroid (BR) related pathways. *CYP90D1* (Cf_f49903) and *CYP90C1* (Cf_f56973) emerged as candidate genes related to stamen length differentiation in *C. fistula*. This study not only helped elucidate the developmental and genetic framework of heteranthery in *C. fistula* but also provided new insights for exploring floral organ evolution in leguminous plants.

## 1. Introduction

During the evolution of angiosperms, diverse morphological traits of flowers have evolved under selective pressure acting on male functions, specifically the dispersal of pollen to receptive stigmas [1,2,3,4]. In most angiosperms, the morphology, size, and color of stamens within the same flower are generally uniform, which is referred to as isomorphic stamens. As direct participants in male function, the morphology and structure of stamens exhibit certain variations under this selective pressure. Significant differentiation is observed among the stamens within a single flower of some angiosperms, a phenomenon known as heteranthery, which has intrigued many researchers ever since the age of Darwin.

Heteranthery has independently arisen on numerous occasions across diverse angiosperm lineages. This floral trait polymorphism has been documented in at least 20 families spanning 12 orders [5,6]. This form of somatic polymorphism is a floral adaptation that functions to mitigate fitness costs associated with pollen consumption by pollinators in species lacking nectar, and in which pollen is the primary floral reward for bees [7,8,9].

Early studies on heteranthery primarily focused on describing floral characteristics and pollinator behaviors (e.g., [10,11]). Based on observations of stamens in Melastomataceae and communications with the German naturalists Fritz and Hermann Müller, Darwin proposed the “division-of-labor” hypothesis for heteranthery [12], that one set of stamens serves to meet pollinators’ nutritional needs by providing pollen as food (“feeding stamens”), while another set ensures the safe transfer of pollen to the stigma (“pollination stamens”). Despite Darwin’s “enormous labor” [12,13] and the extensive work by subsequent researchers on multiple heterantherous groups (e.g., *Commelina coelestis*; *Solanum rostratum*; *Tripogandra grandiflora*; *Senna didymobotrya*) [10,14,15,16], this hypothesis long remained without effective validation until the 21st century. Making use of differentiation in pollen exine ornamentation, Luo et al. (2008) provided the first unequivocal experimental support for Darwin’s division-of-labor hypothesis explaining stamen dimorphism in *Melastoma malabathricum* [8]. In the following years, the functional and evolutionary adaptation of heteranthery has been explored in various plant taxa [7,17,18,19]. However, the developmental mechanisms and molecular regulation underpinning this floral trait remain poorly characterized.

The structure of an organ is the basis of its function. Although the “division of labor” for heteranthery has been experimentally confirmed in species such as *M. malabathricum* [8] and *Solanum rostratum* [9], the developmental mechanisms behind stamen differentiation are still inadequately studied. Tucker’s (1996) research on *S. didymobotrya* proposed that morphological and size differences between “pollination” and “feeding” stamens resulted from asynchronous development of stamen primordia [20]. In *Melastoma* and *Senna bicapsularis*, variations in the initiation time and developmental rates among different stamen groups have also been observed [21]. Nevertheless, key developmental stages determining stamen differentiation are still unclear, and whether different heterantherous taxa share common developmental patterns requires further validation in more species.

Heteranthery serves as an excellent research system for investigating the adaptive evolution of male organs in angiosperms. Despite more than a century of research in this field, critical scientific questions, particularly the mechanistic basis of stamen differentiation, remain unresolved. With advances in sequencing technology, research on model breeding systems such as distyly (e.g., Primulaceae, Turneraceae, Menyanthaceae, Rubiaceae) [22,23,24,25,26] and dioecy (e.g., Ebenaceae, Cucurbitaceae) [27,28] has entered the genomics era. The developmental and molecular regulation of these systems has been elucidated and experimentally validated. In contrast, most studies on heteranthery still focus on macro-level analyses. Recently, Yang et al. (2025) conducted whole-genome sequencing of *Monochoria elata* (Pontederiaceae) and identified genes from the LEAFY, MADS-box, and TCP families as putative regulators governing anther pigmentation, filament elongation, and spatial arrangement, which revealed gene networks underlying heteranthery [29]. However, integrative studies dissecting both developmental trajectories and molecular regulatory pathways of intra-floral stamen differentiation remain limited.

In the large family Leguminosae, heteranthery mainly distributes in several genera of the subfamily Caesalpinioideae, with particularly typical stamen differentiation observed in flowers of *Cassia* and *Senna* [30,31,32,33,34,35,36]. In the recent study, we investigated the development process and genetic basis of heteranthery in *Cassia fistula*, the Golden shower tree, a horticultural species exhibiting significant intra-floral stamen differentiation. This work integrated micromorphological, developmental, metabolomic, and comparative transcriptomic approaches, aiming at elucidating the mechanisms underlying heteranthery formation, thereby providing novel insights into the evolutionary dynamics of floral structure in economic leguminous plants and related angiosperms.

## 2. Results

### 2.1. Floral Biology of Cassia fistula

**Figure 1:**
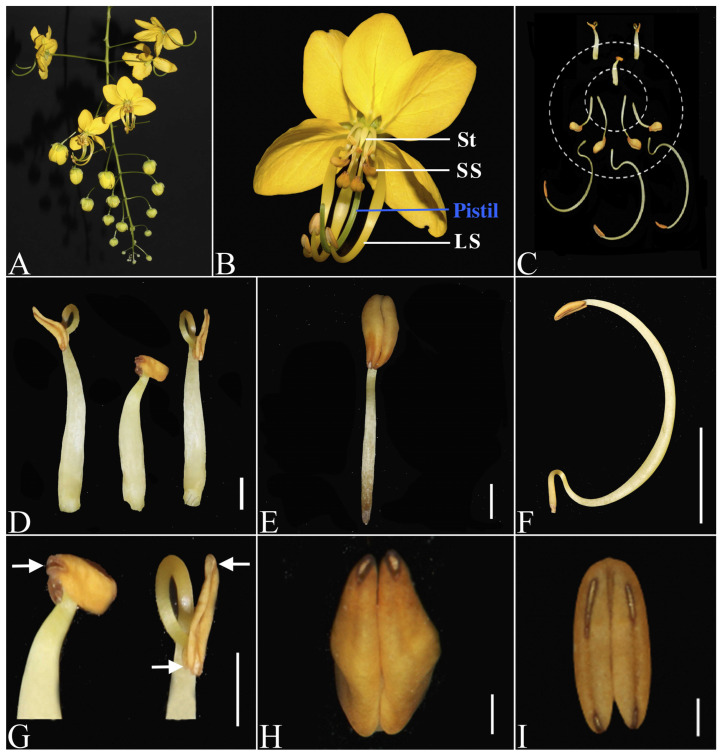
The inflorescence, floral morphology, and stamen differentiation pattern of *Cassia fistula*. (**A**) Raceme; (**B**) Flower, showing the long stamens (LS), short stamens (SS), staminodes (St), and Pistil; (**C**) Distribution of inner and outer whorls of stamens. Dashed circles highlight the two whorls of stamens; (**D**) Staminodes; (**E**) Short stamen; (**F**) Long stamen. (**G**) The anther of the inner staminode (left) with terminal pores (white arrow). The anther of outer staminodes (right) with incomplete longitudinal dehiscence at top and bottom (white arrows); (**H**) The anther of SS showing terminal pores; (**I**) The anther of LS showing incomplete longitudinal dehiscence at top and bottom. Bar = 1 cm in (**F**); 1 mm in (**D**,**G**–**I**); 2 mm in (**E**).

The golden shower tree is an important horticultural plant that produces pendulous racemes with bright yellow flowers (Figure 1A). In South China, *Cassia fistula* typically initiates flowering in early May, with the flowering period extending until September–October. The peak flowering time lasts from June to August. Flowers open at dawn (05:00–06:00), with petals unfurling and coiled stamens/stigmas gradually extending. By 18:00, stamens typically detach. Individual flower longevity is approximately 12 h.

At the study site, primary floral visitors were carpenter bees (*Xylocopa* spp.), including the gray-breasted Carpenter Bee (*X. phalothorax*) and Bamboo Carpenter Bee (*X. dissimilis*). Bees landed on the central short stamens (SS) of the flower when foraging, clasping the anthers tightly against their thorax with their legs while gripping the SS and St filaments with mandibles (Figure S1A,B). Rapid contraction of indirect flight muscles generated vibrations transmitted through the bee’s body to the stamens and pistil, triggering pollen expulsion from the apical pores of the short-stamen anthers, which was later collected by the bees for the larvae’s food. LS pollen dispersed through anther apical slits, adhering to the bee’s dorsal thorax and dorsal abdomen. Concurrently, the stigma at the tip of the curved style contacted the bee’s dorsal part, thereby completing pollination (Figure S1C).

### 2.2. Morphological Differentiation of Androecium

The androecium of *C. fistula* consists of ten stamens arranged in two whorls (Figure 1B,C). The outer whorl is episepalous, comprising three long stamens (LS) and two degenerated stamens (outer staminodes, out-St); while the inner whorl is epipetalous, with four short stamens (SS) and one degenerated stamen (inner staminode, in-St). LS have the longest filaments (30–40 mm), curved in an S-shape, bearing yellow, crescent-shaped anthers that flatten inward and slightly below the stigma (Figure 1B,F,I). Filaments of SS are shorter (6–10 mm long) than those of LS but longer than staminodes, with yellow, elliptical anthers (Figure 1E,H). The outer and inner staminodes have the shortest and straightest filaments, with degenerated anthers (Figure 1D,G).

Another difference in the dehiscence patterns between the two whorls of stamens. For the outer whorl stamens (three LS and two out-St), anthers dehisced through discontinuous longitudinal slits at both apex and base (Figure 1G,I), while anthers of the inner-whorl (four SS and one in-St) dehisced poricidally at the distal end (Figure 1G,H).

### 2.3. Developmental Process of the Three Stamen Sets

The initiation and development of floral organs constitute a continuous process. Following Tucker (1996) [20], the developmental process of floral organs in *C. fistula* can be divided into three stages: (1) Early stage: from the initiation of the floral primordium to the onset of carpel primordium invagination. (2) Mid-stage: from carpel primordium invagination to carpel elongation prior to fusion. (3) Late stage: encompassing the gradual fusion of carpels until the flower is fully matured.

**Figure 2:**
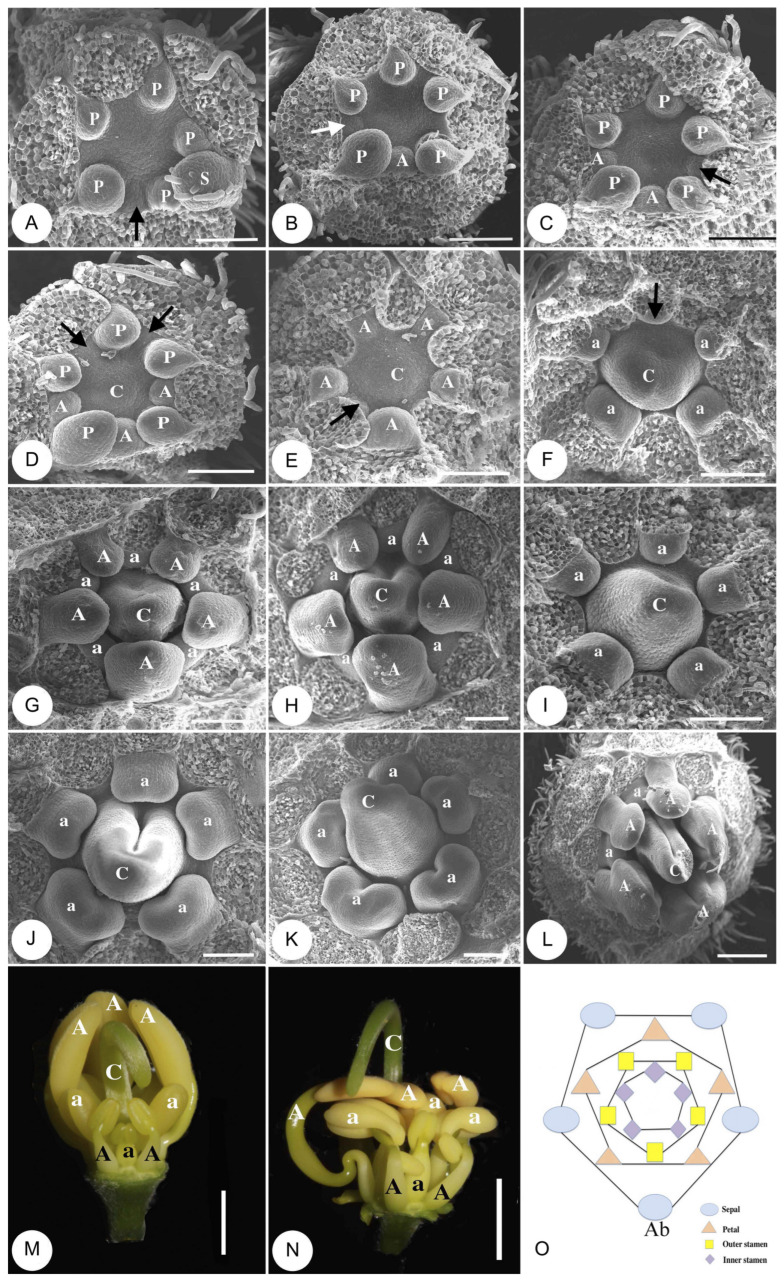
Floral organogenesis and development of *Cassia fistula*. Bracts and bracteoles removed in all figures; sepals removed in (**B**–**N**); petals removed in (**E**–**N**). (**A**) The middle LS primordium arises at the midpoint of the abaxial side, positioned precisely between the first and second petal primordia (“P”), as indicated by the arrow. “S”, sepal primordium. (**B**) The second LS primordium emerges on the left lateral side, as marked by the arrow. (**C**) The third LS primordium emerges on the right lateral side, as marked by the arrow. (**D**) Primordia of the two out-St arise on the adaxial side (arrows). Carpel primordia (“C”) initiate development. (**E**) All five outer whorl (antesepalous) stamen primordia are established (“A”). The arrow points to the initiation site of the first SS primordium. (**F**) Four SS primordia (“a”) are established, and the arrow indicates the primordium of in-St at the central midpoint of the adaxial side. (**G**) The outer whorl (antesepalous, “A”) and inner-whorl (antepetalous, “a”) stamens developed alternately, with the three LS significantly larger than the two out-St. (**H**) Primordia of the three LS become horseshoe-shaped, and epidermal trichomes begin to develop. (**I**). Five stamen primordia of the inner whorl are of similar size. (**J**,**K**) Differentiation between the four SS primordia and the in-St primordium increased; (**L**) The differentiation of anthers and filaments in the outer-whorl stamens has become pronounced, while the development of the three adaxial staminodes (two out-St and one in-St) is markedly delayed. (**M**) Stamen differentiation in the flower bud is ca. 7 mm in diameter. (**N**) Stamen differentiation in a flower bud, ca. 15 mm in diameter. (**O**) Diagram illustrating the arrangement of sepals, petals, outer whorl stamens, and inner whorl stamens. “Ab” indicates abaxial side. Flower buds in (**A**–**L**) are arranged with the abaxial side at the lower side of each figure. Bar = 100 μm in (**A**–**K**); 200 μm in (**L**); 2 mm in (**M**); 5 mm in (**N**).

When all petal primordia had enlarged, the primordium of the middle LS emerged first (Figure 2A), followed sequentially by the primordia of the two lateral LS (Figure 2B,C). Then, primordia of the two out-St form in close succession to complete the whorl (Figure 2D). So, the outer whorl stamen primordia initiated unidirectionally from the abaxial to adaxial side.

The initiation of the inner-whorl (opposite the petals) stamen primordia closely follows that of the outer whorl stamens, also exhibiting a unidirectional developmental sequence. The two abaxial SS primordia sequentially emerge (Figure 2E), followed by the two lateral short stamens, and finally, the adaxial in-St primordium is initiated (Figure 2F).

By the time all inner-whorl stamen primordia have formed, the outer-whorl stamens already exhibit distinct differentiation, with the three abaxial LS primordia significantly larger than the two adaxial out-St primordia (Figure 2G). Subsequently, the three LS primordia initiate differentiation into anthers and filaments. When the apex of the primordia becomes horseshoe-shaped, anther formation commences, accompanied by the growth of epidermal trichomes (Figure 2H). During the development and expansion of the microsporangia, longitudinal grooves have emerged on both sides and the center of the three LS anthers (Figure 2L). In contrast, the two adaxial out-St primordia develop significantly slower than the three abaxial LS primordia, ultimately differentiating into two staminodes with markedly shorter filaments at maturity (Figure 2L–N).

The anther differentiation of inner-whorl stamens initiates relatively late, commencing after the anther differentiation of outer-whorl stamens. Initially, the five inner-whorl stamen primordia develop at a consistent pace with minimal size variation (Figure 2I). As development progresses, the differentiation between the four SS primordia and the single adaxial in-St primordium becomes increasingly distinct (Figure 2J). A prominent groove has emerged along the midline of the SS anthers (Figure 2K), while the development of the in-St is notably delayed (Figure 2K,L, arrows).

From the developmental process, it can be observed that aside from the differences in the initiation timing of development, the LS filaments exhibited a faster overall elongation rate compared to the filaments of SS and St. In the later stages of development, the distinctions among the three stamen groups become increasingly apparent (Figure 2M,N).

The initiation of carpel primordium occurs almost simultaneously with the emergence of outer whorl (antisepalous) stamen primordia. The central region of the floral primordium, initially flat, gradually develops into a dome-shaped structure (Figure 2C–E). Upon the emergence of all five inner-whorl (antipetalous) stamen primordia, the circular carpel primordium is already clearly demarcated (Figure 2F). As carpel depression deepens with stamen differentiation, its incurving margins form a profound cleft (Figure 2J–L), and the initially erect carpel becomes curved within the floral bud (Figure 2M,N). The arrangements of the two whorls of stamens, as well as the sepals and petals in the flower, are illustrated in Figure 2O.

**Figure 3:**
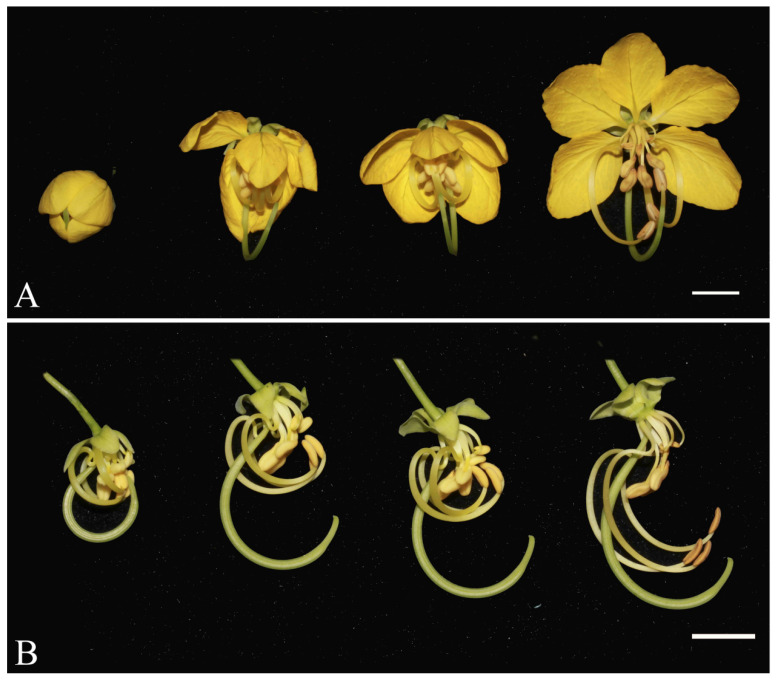
The process of flower opening (**A**) and stamen elongation (**B**), petals removed from *Cassia fistula*. Bar = 1 cm.

During late developmental stages, from the large floral bud (ca. 12 mm in diameter) to anthesis, filament growth in both St and SS of *C. fistula* is largely arrested. In contrast, the filaments of LS and the style continue elongating, forming an S-shaped curvature at the base of the LS filaments. At full bloom, the apex of LS anthers and the stigma are positioned at nearly the same level (Figure 3A,B and Figure S1C), facilitating LS in exercising its function as the “pollination stamen” [37].

### 2.4. Phytohormone Quantification and Comparison

**Figure 4:**
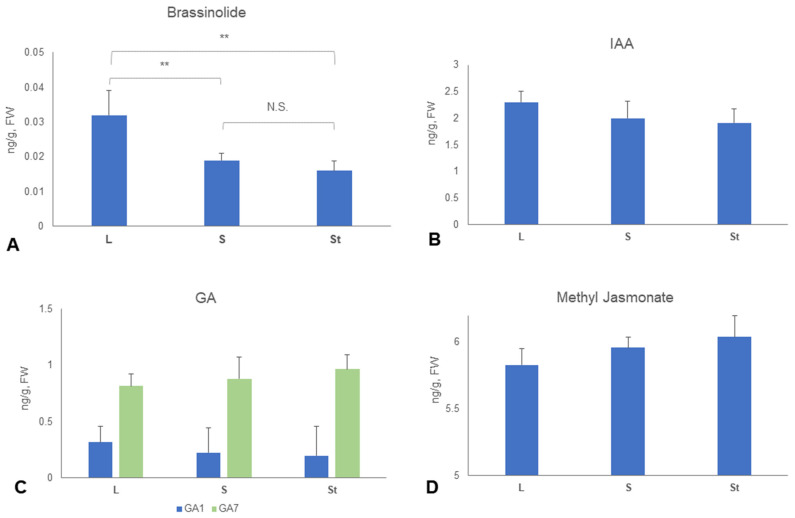
Quantification of important phytohormones in the three types of staminal filaments of *C. fistula*. (**A**) Briassinolide; (**B**) IAA; (**C**) GA; (**D**) Methyl jasmonate. ** *p* < 0.01 in Wilcoxon rank-sum test. N.S. Difference not significant.

Crucial hormones involved in plant organ growth were quantified in the filaments of different stamen types. Brassinolides (BR) promote cell elongation by modulating cytoskeletal organization and cell wall loosening (26). The mean concentration of BR in LS filaments (0.032 ± 0.007 ng/g, FW) was significantly higher than that in SS (0.019 ± 0.002 ng/g, FW) or St (0.016 ± 0.003 ng/g, FW) filaments (Figure 4A). In contrast, similar concentrations were found between LS, SS, and St for IAA (3-indoleacetic acid), GA1 (gibberellic acid 1), GA7 (gibberellic acid 7), and methyl jasmonate (Figure 4B–D).

### 2.5. RNA-Sequencing, Functional Annotation, and Classification

Staminal filaments from LS, SS, and St were subjected to transcriptomic sequencing. We achieved at least 8 Gb clean data for each sample and obtained approximately 293 million pair-end reads with Q30 > 90.0.

After assembly, 145,223 unigenes were obtained for the staminal filament samples. With the aid of the eggNOG-mapper (v.2) pipeline [38], protein sequences of unigenes were searched against different databases, including Swissprot, PFAM, eggNOG, GO, and KEGG for functional annotations. There are 72,815 unigenes that have been annotated in one or more databases (Table 1).

A total of 27,609 unigenes were matched to 128 KEGG pathways for the filament samples. Among them, the pathways “Carbon metabolism (ko01200)”, “Biosynthesis of amino acids (ko01230)”, and “Ribosome (ko03010)” ranked among the top three, having the largest number of annotated genes. Additionally, numerous unigenes were mapped to pathways such as “starch and sucrose metabolism (ko00500)”, “Protein processing in endoplasmic reticulum (ko04141)”, and “Plant hormone signal transduction (ko04075)”, indicating active biosynthesis and metabolic activities (Figure S2).

For the filament samples, 42,783 unigenes were assigned one or more GO terms. Regarding the BP category (Biological Processes), a significant number of genes were associated with the terms “oxidation-reduction process” (GO: 0055114; 3351 unigenes) and “protein phosphorylation” (GO: 0006468; 1956 unigenes). “ATP binding” (GO:0005524; 5625 unigenes) holds the highest representation in the Molecular Function (MF) category, and “integral component of membrane” (GO:0016021; 3740 unigenes) is the most frequently occurring GO term in the Cellular Components (CC) category (Figure S3).

### 2.6. Differential Expression and Enrichment Analysis of Unigenes

Gene expression in filaments and anthers between different stamen types was compared to screen for genes potentially associated with the phenotypic differences during stamen development. For filaments, the largest number of DEGs (168) is found between LS and St, followed by the comparison between LS and SS (49). Only 16 DEGs were identified between SS and St filaments. The number of DEGs was a bit greater than that reported in Yang et al. (2025) [29], which identified 15 DEGs between pollinating and feeding anthers across three developmental stages.

Between LS and St filaments, KEGG pathways “Plant hormone signal transduction” (ko04075), “Sesquiterpenoid and triterpenoid biosynthesis” (ko00909), and “Metabolic pathways” (ko01100) were significantly enriched (*Q*-value < 0.05). Many genes involved in these pathways were related to BR biosynthesis or auxin metabolism, including *CYP90D1* (Cf_f49903), *CYP90C1* (Cf_f56973), *YUCCA* (Cf_f57541), and *AUX28* (Cf_f36862). “Sesquiterpenoid and triterpenoid biosynthesis” (ko00909) was the only significantly enriched pathway between LS and SS filaments, while no significantly enriched pathway was identified for the SS-St comparison.

**Figure 5:**
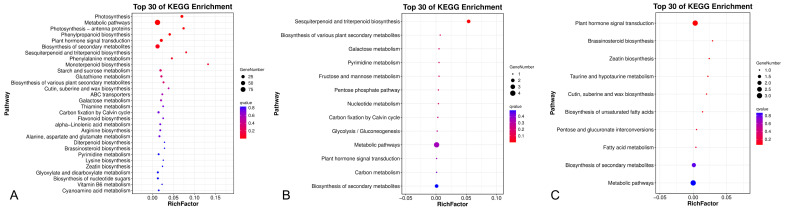
KEGG pathway enrichment analysis for DEGs in the comparisons of LS vs. St filaments (**A**), LS vs. SS filaments (**B**), and SS vs. St filaments (**C**).

Results of the KEGG enrichment analysis are shown in Figure 5 and Figure S4.

### 2.7. Screening of Genes Potentially Associated with Heteranthery

Based on the gene enrichment and phytohormone analysis, we primarily focused on the expression patterns of BR-related genes.

**Figure 6:**
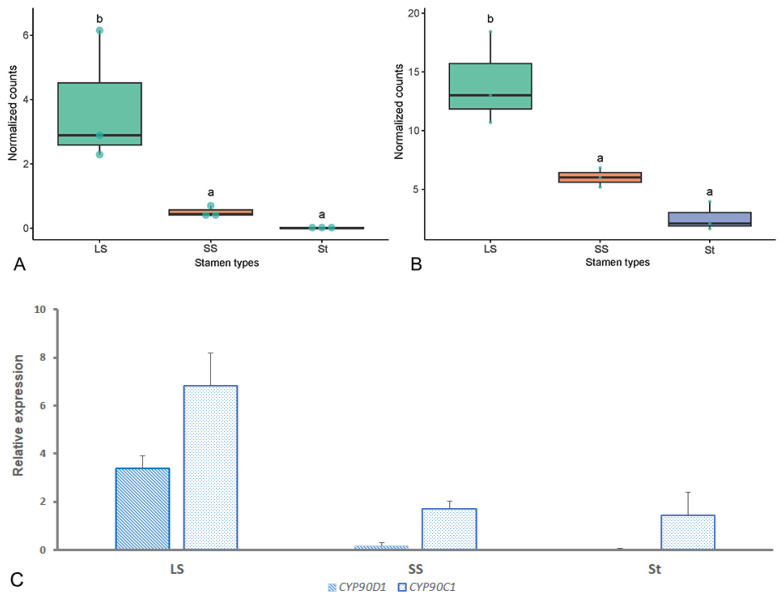
Differential expression of unigenes *CYP90D1* (Cf_f49903) (**A**), *CYP90C1* (Cf_f56973) (**B**), and their expression levels tested by qRT-PCR in three types of stamens (LS, SS, St) of *Cassia fistula*. Different letters (a and b) represent significant differences with Log2(FC) > 1 and FDR < 0.05. Error bars in (**C**) indicate the S.E. of three biological replicates.

DEGs *CYP90D1* (Cf_f49903) and *CYP90C1* (Cf_f56973) are key genes involved in BR signaling. *CYP90D1* plays a critical role in the brassinosteroid (BR) biosynthetic pathway and growth regulation. *CYP90D1* (Cf_f49903) was expressed significantly higher in LS filaments compared to SS and St filaments (Figure 6A). The expression values (FPKM) of Cf_f49903 were lower than one in SS filaments, and <0.1 in the St filaments. *CYP90C1* acts downstream of *CYP90D1*. Its homolog, Cf_f56973, showed much higher expression in LS filaments than St filaments (Figure 6B). qRT-PCR was performed to verify the expression levels of candidate DGEs. The expression pattern of *CYP90D1* (Cf_f49903) and *CYP90C1* (Cf_f56973) in LS, SS, and St filaments is shown in Figure 6C, which is consistent with the RNA-Seq data in general.

### 2.8. Protein–Protein Interaction Network Analysis

PPI networks were constructed to illustrate the biological interactions of the genes of interest in greater detail. Expressions were compared between different stamen types, and unigenes with FoldChange (FC) ≥ 2 in any replicates were selected to create a gene set (Supplementary Materials). Then, protein sequences of these unigenes were mapped to the STRING database, and the generated network was exported to Cytoscape for further analysis. The PPI subnetwork was composed of 1084 nodes and 2420 edges (Figure S5).

BiNGO (v.3.0.5) implemented in Cytoscope was used to assess over-representation of GO categories in the network. “Hormone biosynthetic process” (GO: 0042446), “brassinosteroid biosynthetic process” (GO: 0016132), “brassinosteroid metabolic process” (GO: 0016131), and “flower development” (GO: 0009908) were all significantly enriched (FDR < 0.05), in which *CYP90D1* (Cf_f49903) and *CYP90C1* (Cf_f56973) were included. The related GO term network is illustrated in Figure S6.

## 3. Discussion

As a complex floral trait, heteranthery reflects the high adaptability and evolutionary potential of male organs in angiosperms, holding significant importance for exploring the evolution of breeding systems and floral structural diversity. Although this special floral adaptation has intrigued scientists for over one hundred years, the developmental basis and molecular mechanisms are still underexplored for most heterantherous groups. In the present study, we investigated the development process of different stamen types in *C. fistula*, identified potential gene networks associated with the intra-floral stamen differentiation through transcriptomic and metabolomic analyses.

Morphological differentiation is a hallmark of heteranthery. In *C. fistula*, the androecium displays pronounced structural divergence among its three stamen sets, primarily represented by filament length difference (Figure 1B,C). This phenomenon aligns with observations in other heterantherous taxa, such as *Lagerstroemia indica*, *Senna bicapsularis*, *S. wislizeni*, and *Commelina coelestis* [7,14,35,36,39]. However, the developmental mechanisms underlying filament length variation remain poorly understood across these systems.

Endress (1997) hypothesized that temporal shifts in stamen initiation—specifically, the “centrifugal” developmental pattern in *Dillenia alata*—could lead to asynchronous maturation between outer and inner stamen whorls, thereby contributing to heteranthery [40]. This model provides a plausible framework.

Our data demonstrated that, in *C. fistula*, asynchronous development not only exists between the outer and inner whorls of the stamen but was also detected within the same whorl. The outer whorl stamen preceded the inner whorl stamen in primordia initiation (Figure 2B–H). In the outer whorl, the middle LS initiated first, and then the two lateral LS primordia emerged (Figure 2B–D). The two out-St formed last (Figure 2G,H). In the inner whorl, the two abaxial SS primordia sequentially emerged, followed by the two lateral short stamens, and finally, the adaxial in-St primordium initiated (Figure 2E,F). Therefore, primordia development in both whorls exhibited unidirectional patterns, from the abaxial side to the adaxial side. Tucker (1996) found that in the congenera species *C. javanica*, the abaxial stamens emerged first, showing a similar pattern with *C. fistula* [20]. Marazzi and Endress (2008) also observed the unidirectional initiation order for both stamen whorls in *Senna* species [35].

But Singh and Sharma (1978) have reported the stamens of *C. fistula* initiated “alternatively” in the two whorls [41]. Our result is consistent with Tucker (1996) [20], suggesting the descriptions in Singh and Sharma (1978) [41] might be inaccurate.

The differentiation of LS and St started early at the primordia stage. The three abaxial LS primordia were significantly larger than the two adaxial out-St primordia when carpel depression deepens (Figure 2F–H). Elongation of the LS and the style continued during the development of the floral buds, resulting in comparable lengths in open flowers. This arrangement facilitated the LS anthers and the stigma contacting the dorsal part of the bee’s body and completing pollination (Figure S1C). A similar pattern could also be observed in heterantherous clades *Melastoma* [7,8], *Senna bicapsularis* [7], and *Solanum rostratum* [9], where anther tips of the long stamens (“pollination stamens”) and the stigma contacted the bee’s abdomen when they visited the flowers, in good agreement with the “division of labor” hypothesis [8,9].

The morphological differentiation and “division of labor” for heteranthery are primarily determined by the length and shape of filaments. Yang et al. (2025) suggested that in *M. elata*, jasmonic acid (JA) and lignin might contribute to filament length and morphological differentiation between feeding and pollinating stamens [29]. Luo et al. (2016) reported *ARFs* (Auxin Response Factors) and *GA20ox* (Gibberellin 20-oxidase) potentially regulated stamen length in *S. bicapsularis* [42]. In *C. fistula*, either the IAA, GA, or methyl jasmonate levels differed little among the filaments of long stamens, short stamens, and staminodes (Figure 4). Similar trends have been reported in *M. elata*, where the IAA content was nearly identical in the two anther types [29]. Our analysis reveals that brassinolides (BRs) showed significantly higher concentration in LS filaments compared to SS or St filaments (Figure 4), suggesting a potential correlation of BR content with filament length differentiation in *C. fistula*.

BRs play a pivotal role in regulating cell elongation and organ growth in plants. In *Arabidopsis*, BR-deficient mutants (e.g., *bri1*, *det2-1*) show shortened hypocotyl due to reduced cell size. Additionally, application of BRs to the mutants significantly restored organ elongation by inducing *CESA* genes for cellulose synthesis [43]. In distylous species, the formation of short-styled morphs has been demonstrated to be associated with S-locus genes that degrade BRs in many lineages (e.g., Primula, Gelsemium, Linum, and *Nymphoides*) [26,29,44,45]. Wang et al. (2023) [46] revealed that BRs promoted stamen filament elongation by activating BZR1-family transcription factors (BFTFs), which directly upregulated katanin-encoding genes (e.g., *KTN1*, *KTN80s*). Mutants that lack functional katanin (e.g., *lue1*, *ktn80.1234*) or BFTFs (e.g., *qui-2*) exhibit stunted filament growth due to impaired microtubule dynamics.

Although BRs have important functions in regulating organ elongation across plant species, their potential involvement in the development of heteranthery remains unexplored in the current literature [29,42]. BR concentration was significantly higher in LS filaments than in SS or St filaments of *C. fistula*. The endogenous BR content often exhibits considerable variation across different plant species and tissue types. Although the BR concentration detected in the filaments of *C. fistula* was relatively low, it falls within the range (0.01–0.1 ng/g, FW) reported in previous studies [47,48], potentially implying tissue-specific regulatory mechanisms. Our transcriptomic data revealed that several phytohormone-related KEGG pathways, such as “Plant hormone signal transduction” (ko04075) and “Sesquiterpenoid and triterpenoid biosynthesis” (ko00909), were significantly enriched in the comparison of LS and St filaments in C. fistula (Figure 5, Figures S4 and S5). GO category analysis further demonstrated that “brassinosteroid metabolic process” (GO: 0016131) and “brassinosteroid biosynthetic process” (GO: 0016132) were both over-represented in the PPI network (Figure S6). *CYP90D1* (*Cf_f49903*) and *CYP90C1* (*Cf_f56973*) were included in these pathways and GO terms. *Cf_f49903* was expressed significantly higher in LS filaments compared to St (log2FC = 8.25, FDR < 0.05) or SS (log2FC = 2.96, FDR < 0.05) filaments (Figure 6), i.e., showed an expression ratio of LS:SS:St = 378:51:1. *CYP90D1* plays a critical role in the BR biosynthetic pathway and growth regulation. As a cytochrome P450 enzyme, it is essential for maintaining BR homeostasis, as demonstrated by severe little person phenotypes in *CYP90D1* mutants due to BR deficiency [49,50]. *CYP90C1* acts downstream of early BR biosynthetic enzymes such as *CYP90D1*, ensuring the progression of BR synthesis from sterol intermediates to fully active brassinolide. Both CYP90C1 and CYP90D1 catalyze C-23 hydroxylation of 22-hydroxylated BR intermediates, indicating their roles as functionally redundant C-23 hydroxylases [51,52]. Genetic evidence has been found in *Arabidopsis* that single mutants (*rot3*/*cyp90c1* or *cyp90d1*) showed mild phenotypes (e.g., reduced leaf elongation), while the *cyp90c1 cyp90d1* double mutant exhibited severe dwarfism and male sterility due to rudimentary filaments [50]. The homolog of *CYP90C1* in *C. fistula*, Cf_f56973, shows much higher expression in LS filaments than St filaments (log2FC = 4.82, FDR < 0.05), while only slightly higher than SS filaments (log2FC = 1.00, FDR = 0.9) (Figure 6). This expression pattern suggests *CYP90D1* takes the primary role in regulating filament length, and the low expression of *CYP90C1* might have contributed to the degeneration of St.

## 4. Materials and Methods

### 4.1. Plant Material and Floral Biology Study

*Cassia fistula*, commonly known as the golden shower tree, is native to Southeast Asia and widely distributed in the tropical and subtropical regions of Asia. It is the national flower of Thailand, with great horticultural and economic values. The wood is dense and durable, making it suitable for use as posts or bridges. Additionally, the fruits (pods), bark, and leaves are utilized for medicinal purposes in certain regions.

Samples were collected from mature *C. fistula* trees in Guangdong province. Normally developed and fully opened flowers were randomly selected, and floral characteristics such as flower symmetry, style and stigma morphology, and nectar secretion were examined and recorded. Specifically, attentions were paid to the differentiation pattern of stamens, including the structure, length, and color variations among stamen groups.

### 4.2. Stamen Morphogenesis and Development

Flower buds of different development stages were collected. Some fresh flower buds were dissected and photographed under a stereoscope (SMZ25, Nikon, Tokyo, Japan), and others were fixed in 4% glutaraldehyde solution, vacuumed, and then stored at 4 °C.

Samples for SEM studies were prepared following Luo et al. (2009) [7] and observed with the scanning electron microscope (JSM-6360LV; JEOL, Tokyo, Japan). Images were digitally recorded, and morphological data were analyzed with the aid of software Image Pro Plus (v. 6.0).

### 4.3. Phytohormone Measurements and Analysis

To examine phytohormones potentially involved in filament elongation, fresh filaments from the long, short, and degenerated stamens were collected from three trees and frozen immediately in liquid nitrogen. Mixed-stage samples were weighed and freeze-dried [26]. Extraction, purification, and quantification of BR and other important hormones were performed by Biomarker Technologies Corporation (Beijing, China) according to the company’s protocol, employing the UPLC-MS (Ultra Performance Liquid Chromatography-Mass Spectrometry) system (Thermo, Waltham, MA, USA).

### 4.4. RNA Extraction, Library Construction, and Sequencing

Comparative transcriptomic profiling was conducted to examine the genes and metabolic pathways potentially involved in the differentiation of filaments, anthers, and pollen grains. As the elongation of filaments is a continuous process, filaments from small flower buds (ca. 6 mm in diameter) to fully opened flowers were collected and mixed for each stamen type. There are nine filament samples in all.

Total RNA was extracted with the HiPure Universal RNA Mini Kit (Magen, Guangzhou, China) following the manufacturer’s instructions, then stored at −80 °C until use. RNA quality was assessed by the Agilent Bioanalyzer 2100 system (Agilent Technologies, Santa Clara, CA, USA) before sequencing. RNA samples with RIN (RNA Integrity Number) > 7.0 and OD 260/280 > 2.0 were used for PacBio library preparation, Illumina library construction, and sequencing.

RNA libraries were prepared by the NEBNext^®^ Ultra™ RNA Library Prep Kit for Illumina^®^ (NEB, Ipswich, MA, USA) according to the manufacturer’s instructions. Sequencing was performed on an Illumina Hiseq 2000 platform, and paired-end reads (raw reads) were generated with a length of 150 bp. Raw reads with adaptors or undetermined bases (poly-N) were removed, and reads with low quality (containing more than 50% bases with a *Q*-value ≤ 20) were also discarded.

### 4.5. Gene Functional Annotation

Unigene sequences were aligned by blastx to protein databases NR (http://www.ncbi.nlm.nih.gov/), Swiss-Prot (http://www.expasy.ch/sprot), KEGG (http://www.genome.ad.jp/kegg/), and COG (http://www.ncbi.nlm.nih.gov/COG/) and aligned by blastn to nucleotide databases NT with an e-value < 1.0 × 10^−5^. GO (Gene Ontology) annotation was performed with AmiGO [53] in three ontology categories: Biological Process (BP), Cellular Components (CC), and Molecular Function (MF). KEGG annotation was performed using the KEGG Automatic Annotation Server (KAAS) [54], providing additional functional information showing the pathways in which the transcript isoforms are involved.

### 4.6. Gene Expression and Enrichment Analysis

To identify potential transcripts involved in stamen differentiation, clean reads obtained from Illumina sequencing were mapped to the consensus isoforms using Bowtie2 [55]. The RSEM program was employed to estimate the expression abundances of unigenes [56]. FPKM (the number of fragments per kilobase of exon per million mapped fragments) was used to represent the relative expression levels of each transcript. Differential expression analysis was conducted in R with the DESeq2 package (ver. 1.26.0), and the statistical test results (*p*-values) were corrected for multiple testing with the Benjamini–Hochberg false discovery rate (FDR). Sequences were assigned as DEGs if the FDR ≤ 0.05 and FC (fold change) ≥ 2 (log2FC ≥ 1 or log2FC ≤ −1) between two transcriptomes. qRT-PCR (Real-Time quantitative PCR) was employed to verify the expression of certain important DGEs following standard procedures. Relative abundance of transcripts was determined with the 2^−ΔΔCt^ method, using the actin gene as the reference.

GO and KEGG enrichment analyses of DEGs for different stamen types were conducted using the GSEA (Gene Set Enrichment Analysis) pipeline (v. 4.2.3) [57], a second-generation pathway analysis tool providing more accurate and less biased results by utilizing the entire expression data sets [58], implemented on the Omicshare Bioinformatics Cloud Platform (GENEDENOVO, Guangzhou, China).

### 4.7. Protein–Protein Interaction Network Analysis

Protein–protein interactions (PPIs) are fundamental to most cellular biological processes. Recent studies increasingly integrate gene expression profiles with PPI networks to uncover functional insights into candidate genes. DEGs were analyzed using STRING (https://string-db.org/), a comprehensive database cataloging experimentally validated and predicted PPIs across >5000 organisms. Protein sequences of DEGs were aligned against STRING to construct PPI networks, followed by the removal of isolated nodes and self-interactions.

The resulting networks were exported to Cytoscape (v3.9.1) for topological visualization and further analysis. Subcellular localization annotations for network proteins were retrieved from UniProt (https://www.uniprot.org/) and integrated into Cytoscape. Functional enrichment analysis of Gene Ontology (GO) terms was performed using the BiNGO plugin [59], with statistical significance evaluated via the Benjamini–Hochberg false discovery rate (FDR) correction.

## 5. Conclusions

This study provides an integrated analysis of both developmental processes and molecular mechanisms underlying stamen differentiation in *Cassia fistula*. Our findings reveal that asynchronous development serves as the primary driver for the morphological divergence among the three stamen sets. The initiation of LS preceded both the SS and St. The filaments of LS continued elongation in the following stages until the flowers fully opened, while the development of St and SS filaments was markedly retarded. LS filaments exhibited an overall faster elongation process compared to the SS and St filaments. The cellular basis of filament elongation is an important direction for future studies. Transcriptomic profiling highlighted the potential role of brassinosteroid (BR) biosynthesis and metabolism, with two key genes, *CYP90D1* (Cf_f49903) and *CYP90C1* (Cf_f56973), emerging as candidate regulators related to stamen length differentiation in *C. fistula*. These results help shed light on the developmental and genetic framework of heteranthery in *C. fistula*, and suggest a methodology for exploring floral organ evolution in leguminous plants.

## Figures and Tables

**Table 1:** Number of unigenes annotated in different databases.

Anno_Database	Annotated_Number	300 ≤ Length < 1000	Length ≥ 1000
COG	19,179	7519	6677
GO	42,783	19,494	12,304
KEGG	27,609	12,872	7230
KOG	39,141	17,660	11,647
Pfam	38,903	16,280	14,562
Swissprot	42,912	19,325	13,496
eggNOG	64,952	29,132	18,657
nr	70,647	32,164	19,286
All_Annotated	72,815	32,544	19,325

## Data Availability

Data are contained within the article and Supplementary Materials.

## Supplementary Materials

The following supporting information can be downloaded at: https://www.mdpi.com/article/10.3390/plants14223490/s1, Figure S1: (A) Carpenter bee *Xycolopa phalothorax* is visiting the flower of *Cassia fistula*. (B) Filaments of SS and St have been bitten by *Xycolopa*, showing the dark necrotic marks. (C) A carpenter bee is buzzing the anthers of SS, solid arrow showing the bee grasping the anthers of short stamens with its appendages, and dashed arrow showing the bee gripping the filaments of SS and St with its mandibles. Circle shows anthers of LS and the stigma touching the dorsal surface of bee’s abdomen; Figure S2: Number of unigenes in the top 15 Annotated KEGG pathways for the filament of *Cassia fistula*; Figure S3: Top ten annotated GO terms for filaments in the Biological Processes (BP), Cellular Components (CC) and Molecular Function (MF) categories, respectively; Figure S4: Circular plots showing enriched KEGG pathways in the comparisons of LS vs. St filaments (A), LS vs. SS filaments (B) and SS vs. St filaments (C); Figure S5. PPI network constructed from selected unigenes of *Cassis fistula*. *CYP90D1* (Cf_f49903), *CYP90C1* (Cf_f56973) (dark pink) and directly related unigenes (light pink) were highlighted; Figure S6: Biological network illustrating phytohormone and floral development related GO terms. Colored circles showing over-represented GO categories (FDR < 0.05) in filament comparisons. “Brassinosteroid biosynthetic process” (GO: 0016132) (dark pink), “brassinosteroid metabolic process” (GO: 0016131) (light pink) are highlighted.
