# Supplementary material for: Integrated Transcriptomic and Developmental Analyses Provide Insights into the Intrafloral Stamen Differentiation in Cassia fistula L
Source: Plants (Basel). 2025 Nov 15;14(22):3490. doi: 10.3390/plants14223490 (PMC12655818; doi:10.3390/plants14223490)
Supplement: Supplementary file 1 [file plants-14-03490-s001.zip › plants-3975323-supplementary.pdf]

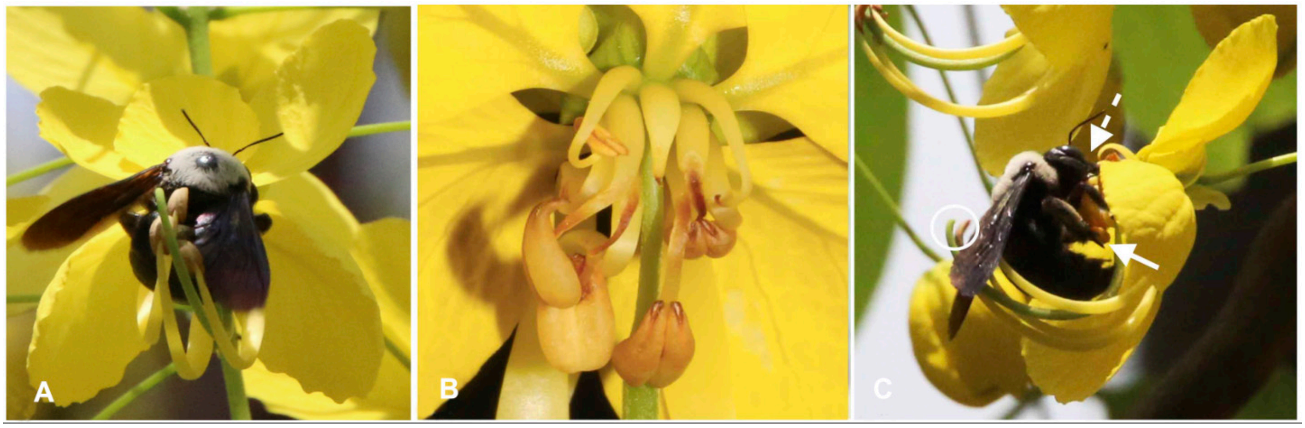

Figure S1. A. Carpenter bee *Xycolopa phalothorax* is visiting the flower of *Cassia fistula*. B. Filaments of SS and St have been bitten by *Xycolopa*, showing the dark necrotic marks. C. A carpenter bee is buzzing the anthers of SS, solid arrow showing the bee grasping the anthers of short stamens with its appendages, and dashed arrow showing the bee gripping the filaments of SS and St with its mandibles. Circle shows anthers of LS and the stigma touching the dorsal surface of bee's abdomen.

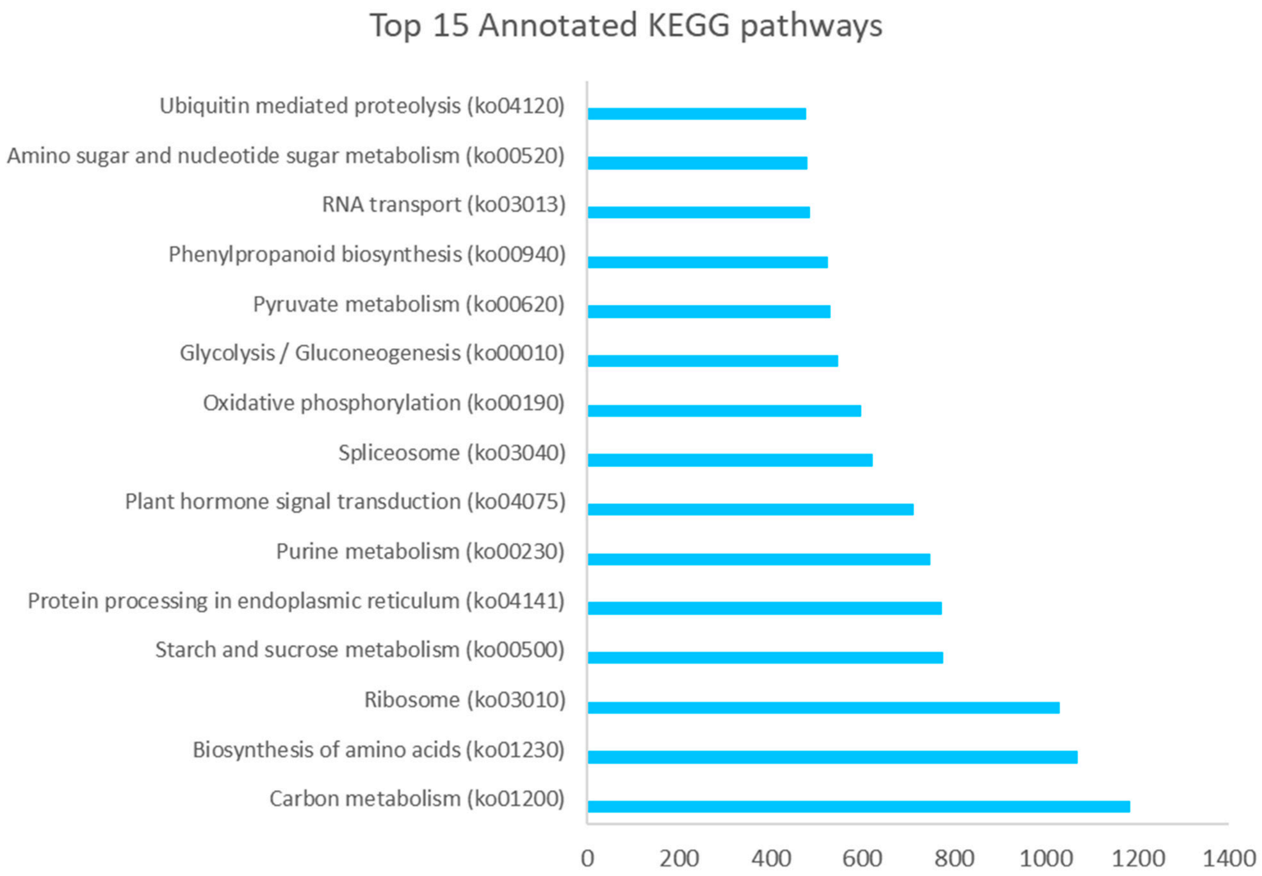

Figure S2. Number of unigenes in the top 15 Annotated KEGG pathways for the filament of *Cassia fistula*.

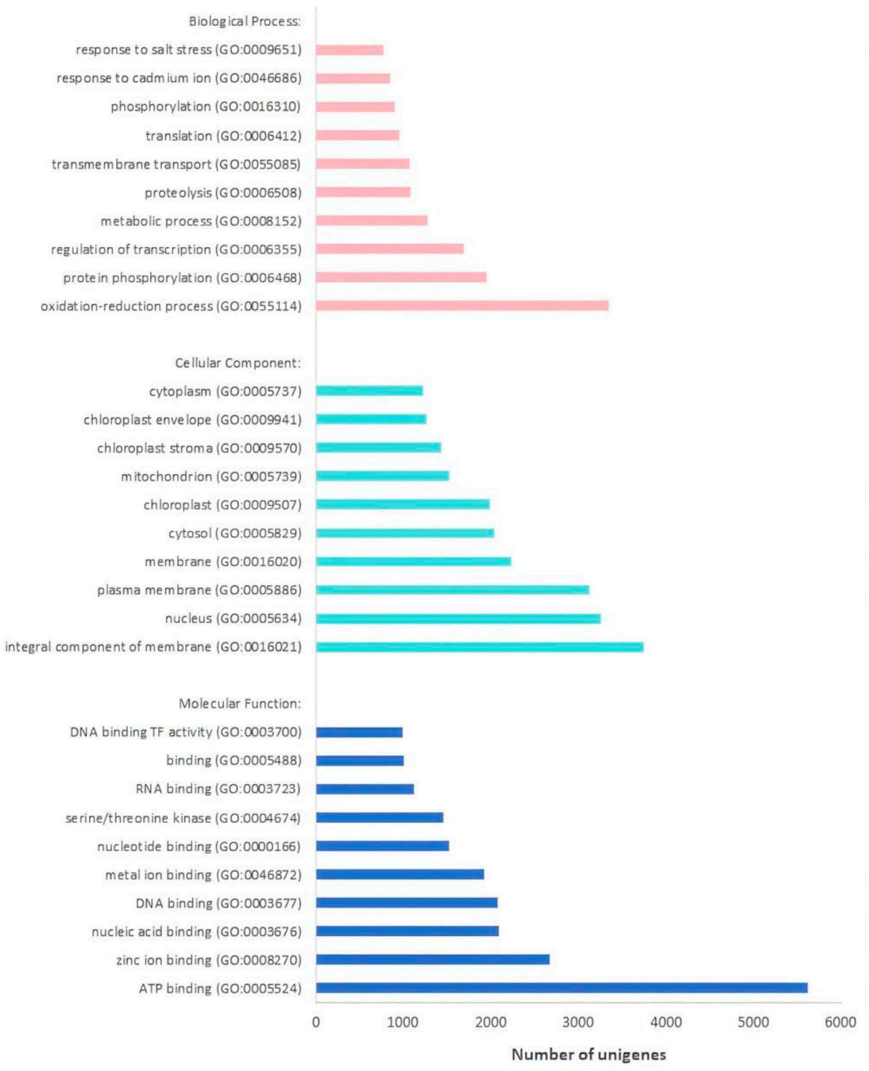

Figure S3. Top ten annotated GO terms for filaments in the Biological Processes (BP), Cellular Components (CC) and Molecular Function (MF) categories, respectively.

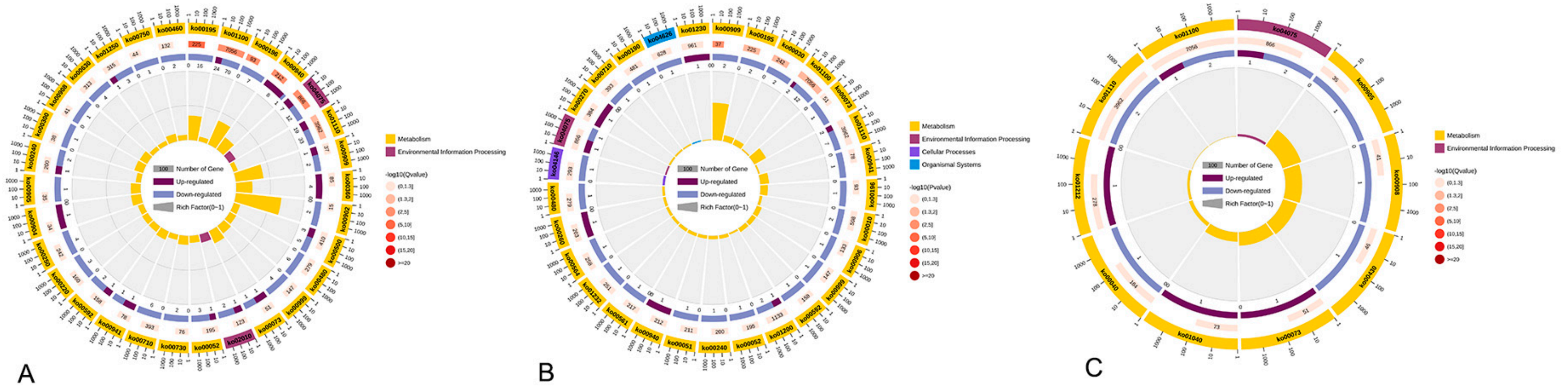

Figure S4. Circular plots showing enriched KEGG pathways in the comparisons of LS vs. St filaments (A), LS vs. SS filaments (B) and SS vs. St filaments (C).

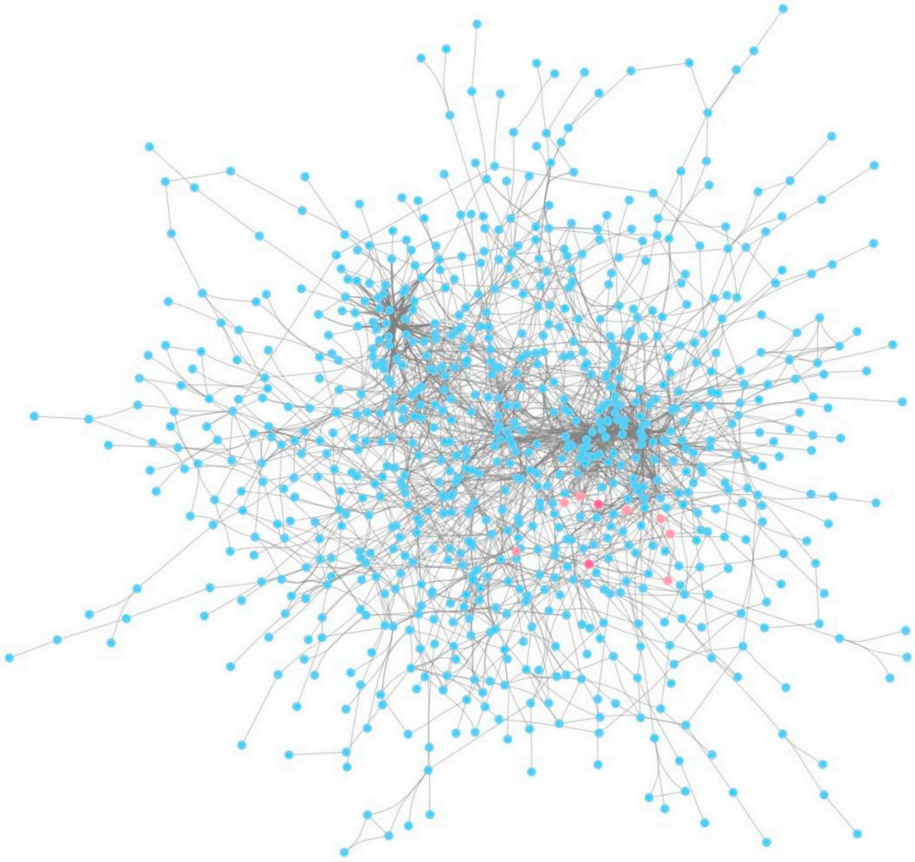

Figure S5. PPI network constructed from selected unigenes of *Cassis fistula*. *CYP90D1* (Cf_f49903), *CYP90C1* (Cf_f56973) (dark pink) and directly related unigenes (light pink) were highlighted.

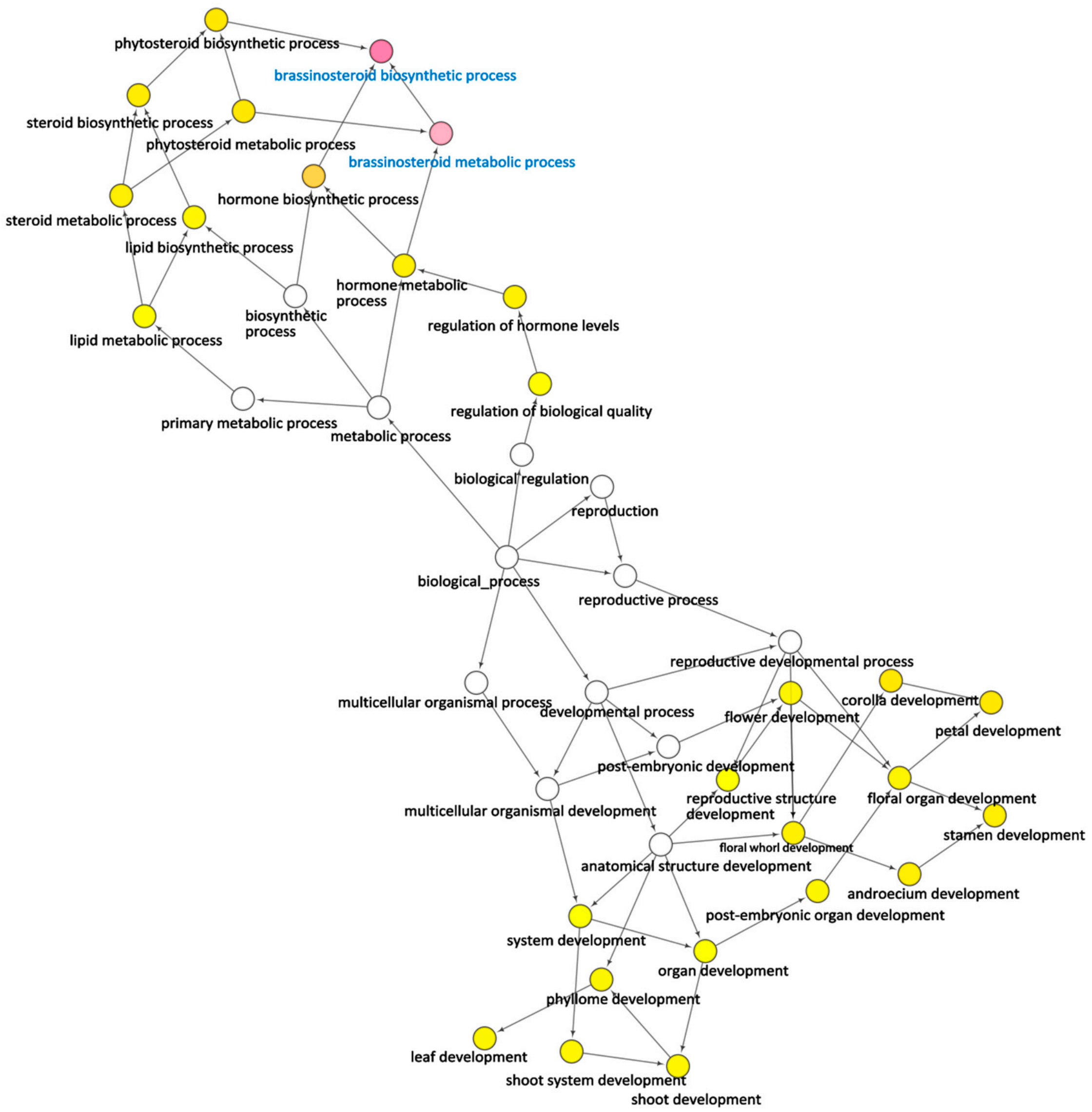

Figure S6. Biological network illustrating phytohormone and floral development related GO terms. Colored circles showing over-represented GO categories (FDR<0.05) in filament comparisons. "Brassinosteroid biosynthetic process" (GO: 0016132) (dark pink), "brassinosteroid metabolic process" (GO: 0016131) (light pink) are highlighted.
